# Female Gender Remains a Significant Barrier to Access Cataract Surgery in South Asia: A Systematic Review and Meta-Analysis

**DOI:** 10.1155/2020/2091462

**Published:** 2020-01-11

**Authors:** Qunru Ye, Yanxian Chen, William Yan, Wei Wang, Jingxian Zhong, Cong Tang, Andreas Müller, Bo Qiu

**Affiliations:** ^1^The Second Clinical College of Guangzhou University of Chinese Medicine, Guangzhou, China; ^2^Jiangmen Xinhui Aier New Hope Eye Hospital, Jiangmen, Guangdong, China; ^3^Zhongshan Ophthalmic Center, State Key Laboratory of Ophthalmology, Sun Yat-Sen University, Guangzhou, China; ^4^Centre for Eye Research Australia, Royal Victorian Eye and Ear Hospital, University of Melbourne, Melbourne, Australia; ^5^WHO Collaborating Center for Prevention of Blindness, Centre for Eye Research Australia (CERA), University of Melbourne, Melbourne, Australia; ^6^Department of Ophthalmology, University of Melbourne, Melbourne, Australia

## Abstract

**Purpose:**

To determine whether the female gender is a barrier for the access to cataract surgery services in South Asia in the last two decades.

**Methods:**

Eligible cross-sectional studies were identified via computer searches and reviewing the reference lists of the obtained articles. The cataract surgical coverage (CSC) by sex based on person and eyes at visual acuity <3/60 and 6/18 is extracted. Pooled odds ratios (ORs) for males receiving cataract surgery in comparison with females were calculated by a random effect model.

**Results:**

Sixteen studies with 135972 subjects were included in the final analysis. The pooled ORs of CSC by sex on a person basis at visual acuity <3/60 and at visual acuity <6/18 were 1.46 (95% CI: 1.23–1.75) and 1.14 (95% CI: 1.05–1.24), respectively. For CSC on a per-eye basis at visual acuity <3/60, the associations were statistically significant, with a pooled OR of 1.40 (95% CI: 1.16–1.70). The values of population attributable risk percentage at a per-person and per-eye basis at visual acuity <3/60 were 6.28% and 7.48%, respectively. Subgroup analyses by design and location types attained similar results as the primary analyses. There was no evidence of publication bias.

**Conclusions:**

The female gender remains a significant barrier for the access to cataract surgery in South Asia. Visual impairment, including blindness, from unoperated cataract, could be reduced by approximately 6.28% with the elimination of gender disparities to access. More efforts are needed to increase eye care service utilization by female population.

## 1. Introduction

In 2010, it was estimated that there were 39 million people blind globally, with 51% being attributed to cataract [1]. In particular, the female population accounts for approximately 60.0% of blindness and 57% of moderate-to-severe visual impairment [2]. South Asia, which accounts for 24.6% of the world's population, has undergone fundamental transitions in society and economic development over the past two decades. Although this region has achieved significant progress in blindness prevention, it still accounts for 32.7% of the world's blind population in 2010 [2]. In addition, the expansion rate of the blinded population was 4 times higher in females than that in males (16.1% versus 3.7%) from 1990 to 2010, which poses significant public health concerns [2, 3]. Cataract remains the leading cause of blindness, which accounts for 41.7% blindness in this region in 2010 [3].

Cataract blindness can be cured by a straightforward and cost-effective procedure. Economic analysis demonstrates that cataract surgery led to substantial improvements in quality of life, increases in income, and alleviation of poverty [4–7]. The financial return on investment of the first-eye cataract surgery was estimated to be 4567% over the 13-year model in the United States [8, 9]. Cataract surgical coverage (CSC) was recommended by the World Health Organization (WHO) for evaluating the accessibility and utilization of cataract surgical services in a given district. It was endorsed by the Global Action Plan (GAP) to assess and monitor the national eye service. CSC indicates the percentage of cataract surgeries and cataract surgical needs of the population, which reflects the actual community level situation.

Several years ago, the WHO and other nongovernment organizations (NGOs) introduced the VISION 2020 program to reduce the burden of vision loss worldwide. Considering the disproportionately high level of blindness in females, concerns over gender inequality were emphasized as the theme of the World Sight Day in 2009: Gender and eye health: equal access to care [5]. In a meta-analysis conducted 2 decades ago, Abou-Gareeb et al. reported that the chance of blindness in developing countries was 1.3 times higher in females compared to males [2, 10]. Previous anecdote meta-analysis indicated that the female gender was a barrier to the access of cataract surgery [11, 12]. However, limited studies were included in these analyses and the state of gender inequalities in access to cataract surgical services in South Asia is not well understood. Therefore, a systematic review and meta-analysis of population-based cross-sectional studies were performed to assess whether the female gender remains a significant barrier to access of cataract surgery in South Asia in the past two decades.

## 2. Materials and Methods

The protocol of this study was registered on the International Prospective Register of Systematic Reviews (CRD42017054426). This systematic review and meta-analysis were performed in accordance with the PRISMA statement (https://www.crd.york.ac.uk/PROSPERO/display_record.php?ID=CRD42017054426&ID=CRD42017054426) [13, 14]. Literature searches, study selection, and data extraction were conducted by two independent reviewers (QY and YC). Any disagreements were resolved by discussion and consulting a third senior reviewer (WW) if needed.

### 2.1. Data Sources and Searches

A systematic literature search was performed in Pubmed, Embase.com, and ISI Web of science from Jan 2000 to Jan 2019. The common keywords related to cataract surgery, cataract surgical coverage (CSC), population-based studies, and country names were used. The search strategy is detailed in Appendix 1. No language restriction was imposed. The Google Scholar Engine, Rapid Assessment of Avoidable Blindness (RAAB) repository (http://raabdata.info/repository/), and WHO website (http://www.who.int/en/) were also used to identify eligible studies. The reference lists of the included articles and related reviews were also screened to identify additional eligible studies.

### 2.2. Study Selection

Studies were included for analysis if they met the following criteria: (1) they were performed after 2000; (2) the study design was population-based or in the form of RAAB, national registry, or national/subnational survey of all populations; (3) the study was conducted in a general adult population; (4) the study reported the number of cataract surgery performed (person/eyes) and the number requiring cataract surgery (person/eyes) at visual acuities of <3/60 (20/400) or 6/18 (20/60), or provided the data needed to calculate them. To eliminate the bias by refractive errors, only the corrected or pinhole visual acuity was used for calculating CSC. For the same population with several studies performed at different times, only the latest one was included. For the same studies with multiple publications, only the most complete was included and the others were referenced for data extraction as needed. Hospital-based studies, studies in special settings such as leprosy villages, meeting abstracts, and studies with insufficient data were not included.

### 2.3. Data Extraction

For each study, the following information was collected: first author's name, year of publication, study design, location, characteristics of subjects, number of people receiving examinations, response rates, CSC by sex based on persons and eyes at different visual acuity cutoff, the number of persons/eyes operated, and the number of persons/eyes needing cataract surgery. CSC can be calculated at a per-person level or a per-eye level based at different visual acuity levels (<3/60, 6/60, and 6/18). For eyes, CSC is calculated as (*a*/(*a* + *b*)) × 100, where *a* = number of (pseudo)aphakic eyes and *b* = number of eyes with operable cataract (BCVA<3/60; <6/60; <6/18). For a person, CSC is calculated as ((*x* + *y*)/(*x* + *y* + *z*)) × 100, where *x* = persons with bilateral (pseudo)aphakia, *y* = persons with 1 (pseudo)aphakic and 1 operable cataract, and *z* = persons with bilateral operable cataract at different VA levels [15]. The two extreme cutoffs (3/60 and 6/18) were used in this study.

Many studies did not report raw data for calculating CSC and its 95% confidence interval (95% CI). To address this, we followed the methodology of previous meta-analysis by Lewallen et al. [11, 12]. For studies without raw data but reporting CSC by sex and their 95% CI, we calculated backwards to estimate the denominator (N), which equals to the sum of operated persons (eyes) and the number of persons (eyes) requiring cataract surgery. The following function was used: *N* = 1.96^2^ × 100 × CSC × (100 − CSC)/(CSC − lower 95% CI)^2^. The number of operated persons (eyes) and the number of persons (eyes) requiring cataract surgery were calculated based on *N* and CSC at a specific level. For studies providing both raw data and CSC with 95% CI, the former was used in the meta-analysis.

### 2.4. Assessment of Risk and Bias

The methodological quality of the included studies was assessed using a risk and bias tool in prevalence studies [16]. It consists of ten items related to the representativeness of the sample, the sampling technique, risk of nonresponse bias, the data collection method, the case definition, the ascertainment toll, and the statistical method. Each item was scored 1 or 0 to represent a “low risk of bias” or “high risk of bias.” The total score ranged from 0 to 10 with 0–3 for high risk, 4–7 for moderate risk, and 7 and greater for low risk.

### 2.5. Data Synthesis and Statistical Analysis

Three methods were used to summarize the impact of the female gender on CSC. First, for each study, the respective CSC for males and females was used to calculate an OR using the formula OR = CSC_male_ × (100 − CSC_female_)/((100 − CSC_male_) × CSC_female_) [11]. Then, the weighted average (mean) OR of these ORs was calculated based on sample sizes in each study. Second, a meta-analysis was performed using raw data to calculate the pooled ORs based on person (eyes) at a visual acuity of <3/60 and 6/18. Third, a pooled relative risk was calculated and used to estimate the blindness (visual acuity<3/60) population-attributable risk percentage (ARP) using the formula ARP = *P* × (RR − 1)/[*P* × (RR − 1) + 1], where *P* = proportion of female in adults population, estimated at 55% [11]. Statistic heterogeneity between studies was tested using Cochran's *Q* and *I*^2^ statistics, which was considered significant when *P* < 0.10 and/or *I*^2^ ≥ 50% [17]. A random effect model was used. Subgroup analysis was performed by study design and location type. Sensitivity analysis was also performed using single-study influence analysis. The potential publication bias was assessed using Begg's tests and Egger's test [18]. All analyses were performed using STATA version 12.0 (Stata Corp). A *P* value < 0.05 was considered significant, except where otherwise specified.

## 3. Results

### 3.1. Literature Search

**Figure 1:**
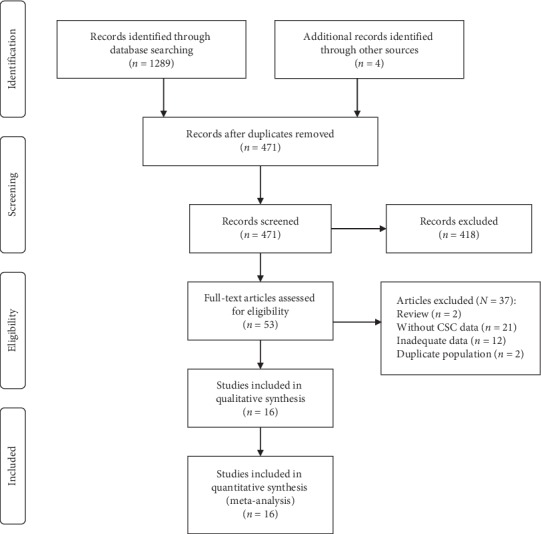
Diagram of study selection; CSC = cataract surgical coverage.

Figure 1 shows the process of the study selection. After the title and abstract review, 53 potentially eligible studies underwent full-text review. Thirty-seven papers were excluded: 2 due to review design, 21 did not have CSC data, 12 had inadequate CSC data, and 2 had overlapping populations with included studies. Sixteen independent studies with a total of 135972 subjects were included in the final analysis [19–34].

### 3.2. Study Characteristics

The characteristics of the included studies are presented in Table 1. Of which, 14 studies were published in peer-reviewed journals, while 2 were unpublished [32, 33]. One study was national, while others were regional. The number of examined people ranged from 1076 to 39908. Two studies were conducted in Bangladesh, one in Bhutan, four in India, five in Nepal, and four in Pakistan.

### 3.3. Methodological Quality

All 16 studies were categorized as having a low bias (Table 2). Eight studies were conducted in single or multiple provinces (6 in rural and 2 in urban) and were not clear if it was representative of the national population. All studies reported the method of participant selection and the random sampling technique. The response rates in all studies were over 75.0% (median 92.25%).

### 3.4. Weighted Mean OR

Table 3 shows the CSC by sex based on persons or eyes at different visual acuity levels. For CSC based on persons at the blind level, the CSC values were higher in males than in females in all except three studies. For studies reporting CSC based on other levels, similar phenomena were observed. The weighted mean OR by sample size was 1.5, 1.1, 1.4, and 1.1 at different levels, respectively.

### 3.5. Meta-Analysis and PAR

**Figure 2:**
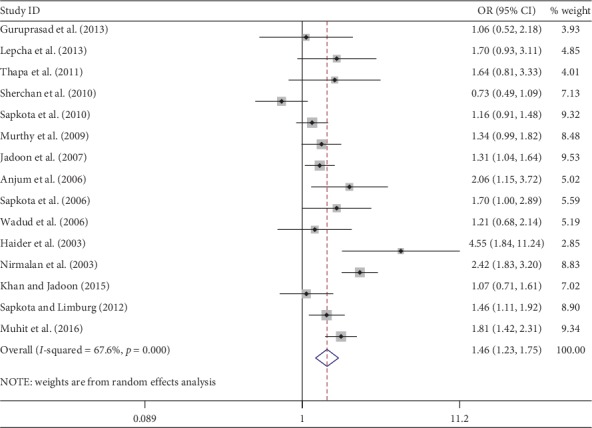
Forest plot showing the meta-analysis of CSC by sex on a person basis at visual <3/60 level. CSC = cataract surgical coverage; OR = odds ratio; CI = confidence interval.

**Figure 3:**
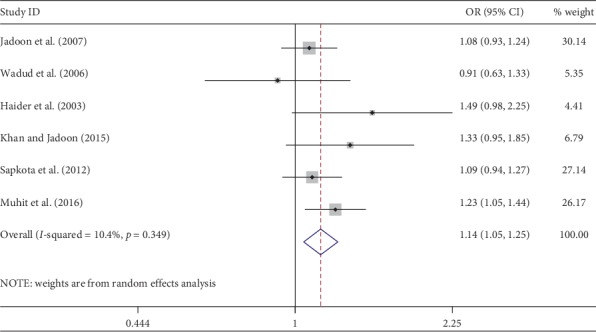
Forest plot showing the meta-analysis of CSC by sex on a person basis at visual <6/18 level. CSC = cataract surgical coverage; OR = odds ratio; CI = confidence interval.

**Figure 4:**
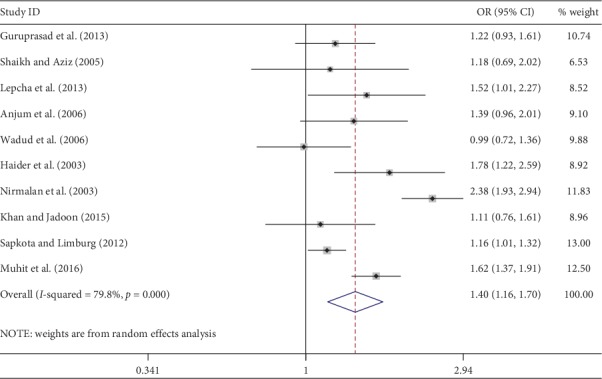
Forest plot showing the meta-analysis of CSC by sex on an eye basis at visual <3/60 level. CSC = cataract surgical coverage; OR = odds ratio; CI = confidence interval.

**Figure 5:**
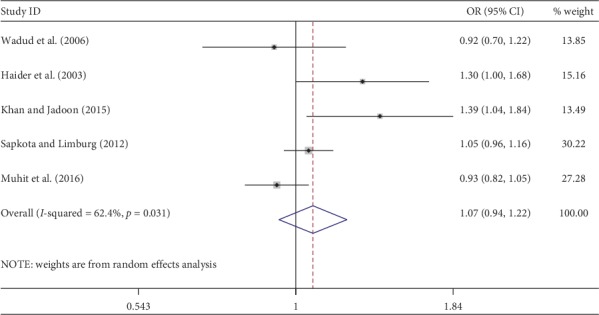
Forest plot showing the meta-analysis of CSC by sex on an eye basis at visual <6/18 level. CSC = cataract surgical coverage; OR = odds ratio; CI = confidence interval.

Meta-analysis was used for 15 studies. The meta-analysis of CSC by sex on a person basis (visual acuity <3/60) showed a pooled OR of 1.46 (95% CI: 1.23–1.75) for males use of cataract surgery compared with females (Figure 2). The heterogeneity was significant (*P* < 0.001; *I*^2^ = 67.6%), and a random effect model was adopted. Results were similar for the meta-analysis of CSC by sex on a person basis at a visual acuity <6/18, with a pooled OR of 1.14 (95% CI: 1.05–1.24) with no significant heterogeneity among studies (*P*=0.349; *I*^2^ = 10.4%) (Figure 3). Regarding the CSC on an eye basis at visual acuity <3/60, the associations were statistically significant, with a pooled OR of 1.40 (95% CI: 1.16–1.70) (Figure 4). Only 5 studies were available for meta-analysis of CSC on an eye basis at visual acuity <6/18, and the pooled OR was not significant due to substantial heterogeneity among studies (Figure 5).

**Figure 6:**
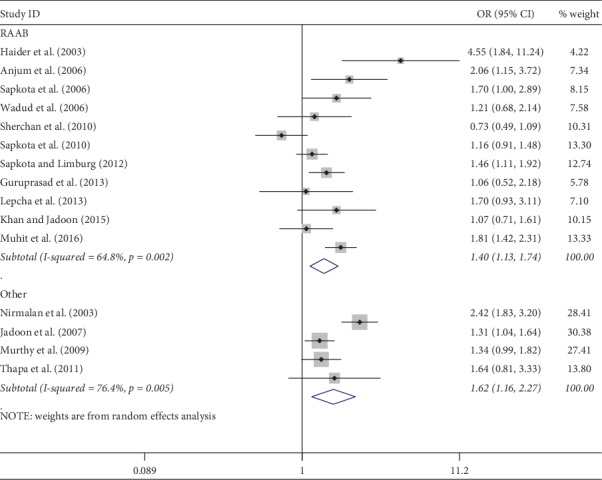
Subgroup analysis of CSC by design on a person basis at visual <3/60 level. CSC = cataract surgical coverage; OR = odds ratio; CI = confidence interval.

**Figure 7:**
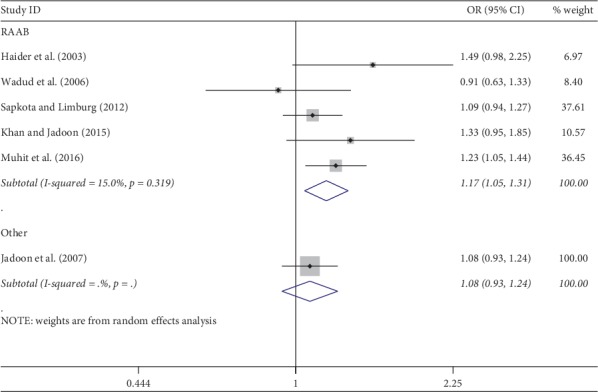
Subgroup analysis of CSC by design on a person basis at visual <6/18 level. CSC = cataract surgical coverage; OR = odds ratio; CI = confidence interval.

**Figure 8:**
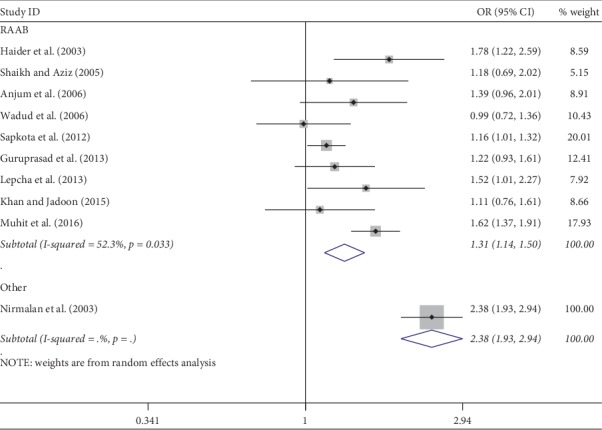
Subgroup analysis of CSC by design on an eye basis at visual <3/60 level. CSC = cataract surgical coverage; OR = odds ratio; CI = confidence interval.

**Figure 9:**
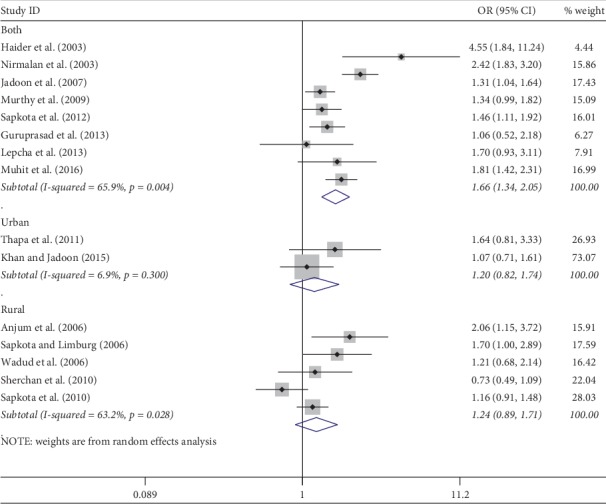
Subgroup analysis of CSC by location on a person basis at visual <3/60 level. CSC = cataract surgical coverage; OR = odds ratio; CI = confidence interval.

**Figure 10:**
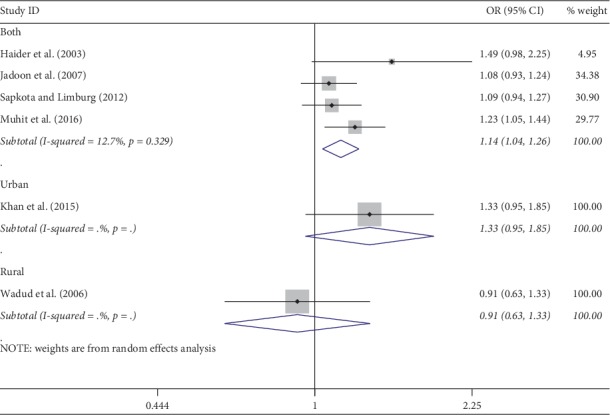
Subgroup analysis of CSC by location on a person basis at visual <6/18 level. CSC = cataract surgical coverage; OR = odds ratio; CI = confidence interval.

**Figure 11:**
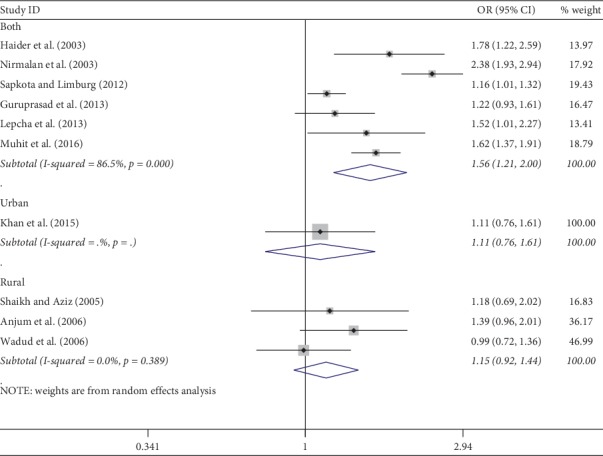
Subgroup analysis of CSC by location on an eye basis at visual <3/60 level. CSC = cataract surgical coverage; OR = odds ratio; CI = confidence interval.

**Figure 12:**
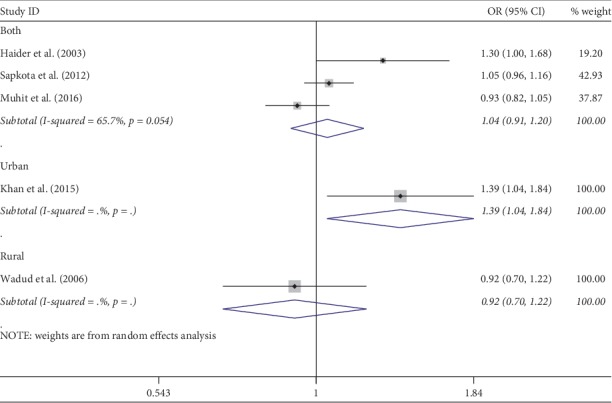
Subgroup analysis of CSC by location on an eye basis at visual <6/18 level. CSC = cataract surgical coverage; OR = odds ratio; CI = confidence interval.

**Figure 13:**
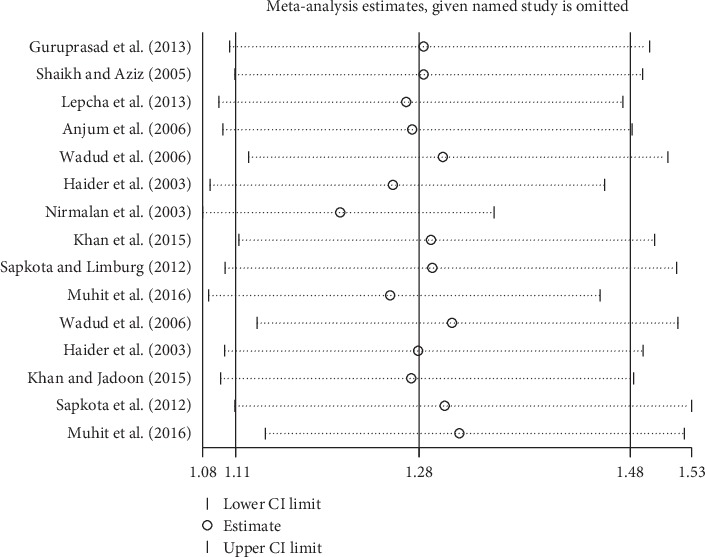
Sensitivity analysis of CSC on an eye. CSC = cataract surgical coverage; CI = confidence interval.

**Figure 14:**
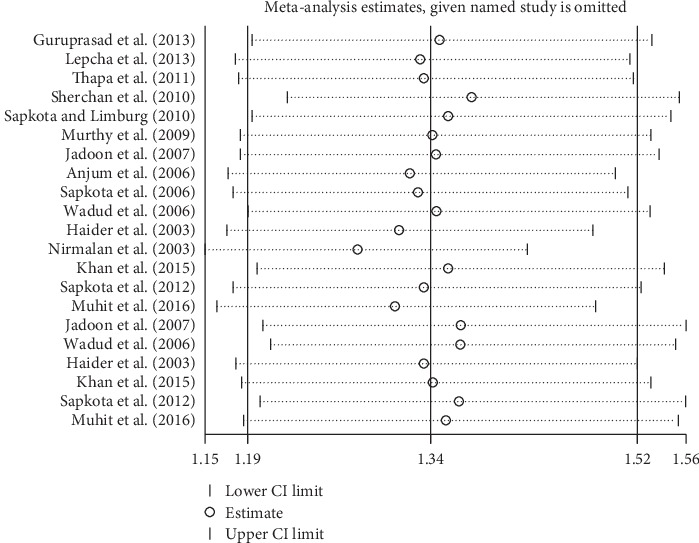
Sensitivity analysis of CSC on a person. CSC = cataract surgical coverage; CI = confidence interval.

Subgroup analyses by design and location types produced similar results as the primary analyses (Figures 6–12). Further sensitivity analyses that omitted one study at a time and calculated the pooled ORs for the remaining studies yielded consistent results (Figures 13 and 14). There was no evidence of publication bias, as indicated by nonsignificant Begg's tests (*P*=0.488) and Egger's tests (*P*=0.173) in persons at VA <3/60. The values of ARP at a person and eye basis were 6.28% and 7.48%, respectively.

## 4. Discussion

This study first provides a summary of gender inequity in CSC in South Asia with 16 eligible including 5 countries. Most studies were performed in subnational regions using the RAAB design. Our results demonstrated that the female gender remains a significant barrier to the access of cataract surgery in South Asia, with males having 1.46 odds of receiving cataract surgery compared to females per-person at blind VA. A very similar meta-analysis from India has been recently published in the British Journal of Ophthalmology. Prasad et al. reported that CSC was found to be 27% lower in women than men in India, and it could be improved by 13.4% in women if the gender gap in coverage is eliminated [35]. This is also supported by our findings, where women do not receive cataract surgery at the same rate as men do, and closing this gender gap may be a much-needed step.

Although gender inequity remains a significant problem in Nepal; however, it is not necessarily true for all programs. The pooled OR of CSC at blind VA was 1.09 (95% CI: 0.77–1.54) based on 5 studies in Nepal. Sherchan et al. reported that in the past 10 years, blindness prevalence, particularly due to cataract, has decreased and cataract surgical coverage has increased in the Lumbini Zone and Chitwan District. Cataract and other surgical services are equitably distributed by age and sex throughout all districts in the service area [23]. In another example in Nepal, Thapa et al. reported that the cataract surgical coverage was 90.36% and was high in females and illiterate subjects in Bhaktapur [22]. These are all indicators that cataract intervention programs have been successful in these areas.

Our study confirmed and extended previous efforts on the gender disparities in eye disease/service. In 2001, Abou-Gareeb et al. [10] reported that the prevalence of blindness was higher in females compared to males, which did not analyze the cataract blindness. In 2009, Lewallen et al. reported that the pooled OR of CSC was 1.71 (95%CI: 1.48–1.97) based on persons at visual acuity <6/60 [11]. However, these studies were limited by the inclusion of relatively outdated studies, with only a few studies from South Asia, and did not access raw data from their selected studies. Furthermore, the studies only used CSC person data at a visual acuity <6/60 with various methods. In Latin America, Cart et al. [15] reported that gender did not play a significant role in access to cataract surgery. Our results were consistent with the analyses by Lewallen et al. [11, 12]. The strength of this analysis lies in the inclusion of a large number of studies from 5 countries, use of raw data, multiple analyses at different levels, and use of data that reflect current situations with respect to cataract surgery programs and access. Furthermore, we observed progress in alleviating gender inequity. It was estimated that cataract blindness could have been reduced to a rate of 12.5% in 2000 and 10.8% in 2008 if the males and females had the same CSC rates [11, 12]. Our results suggest that this figure may have been reduced to 6.28% in South Asia, indicating persistent efforts aimed at reducing the gender gap in eye care access are warranted.

Possible explanations for the gender inequity in CSC are related to the social, economic, and cultural differences between males and females [12, 36]. The use of cataract surgical services was reported to be associated with the cost of cataract surgery and transportation, awareness of cataract surgery, and community-based education [37]. Females generally had higher illiteracy rates, especially amongst the elderly, reducing access to such information regarding treatment. It has been reported that the literacy of the affected person and their family members was a key predictor of receiving cataract surgery in South India [31]. Females may also have fewer financial resources to pay for eye care and transportation to hospitals compared to their male family counterparts. Various epidemiology studies confirmed the higher prevalence of cataract in females compared to males [10, 38]. The circumstances in South Asia are complex and may be related to work role, education, and economic decision-making power. Further research is needed to better understand these phenomena.

Our results have major implication in planning blindness prevention programs. Equal access to eye care could substantially reduce blindness in South Asian countries. For cataract programs, it was estimated that the proportion of cataract surgery performed in females should be 60%–65% to produce an equal CSC between genders [10]. Action should be taken to reduce the gender gap in CSC to achieve the blindness prevention target set out by the GAP. An anecdotal study in Pakistan showed that the following interventions can effectively address the barrier to cataract surgery: reducing surgical cost, flexibility of service time, reducing the need for accompanying family members, providing transport, and educational outreach [39]. As women have a higher incidence of cataract and longer life expectancy, a higher rate of cataract surgery in female populations should be emphasized in cataract programs. The female cataract surgery account for 59%–66% of all cataract surgery in developed countries such as France and Sweden [40, 41]. Therefore, strategies aimed at increasing eye care service utilization, especially access of cataract surgery by women, are warranted.

This study has several limitations. First, most of the included studies were not designed specifically to assess the gender inequity in access to cataract surgery. The risk and bias of these studies were not evaluated; however, as the majority was of the RAAB format using standardized methodologies and the results were considered to be reliable. Second, CSCs were calculated based on all operated eyes; however, those receiving the surgery may not necessarily have had visual impairment preoperatively. Nevertheless, our findings were robust as sensitivity analysis yielded virtually unchanged results. Third, most studies were performed in subnational areas, and for large countries such as India, substantial variations may exist across regions. Fourth, this study only included five countries in South Asia, including India, Pakistan, Nepal, Bangladesh, and Bhutan. Sri Lanka and Maldives were not included because relevant studies conducted in these two countries were fewer and they did not meet the inclusion criteria. Finally, the CSC does not take surgical quality into account. In a rural Southern Indian population, it was reported that 7.2% of blindness was iatrogenic and attributed to cataract surgery. Gender gaps in CSC and cataract surgical outcomes should be evaluated in further cataract program analyses.

## 5. Conclusions

In summary, the female gender remains a significant barrier to the access of cataract surgery in South Asia. We propose that once the female population achieves an equal CSC compared to males, the prevalence of cataract blindness can be reduced by around 6.28% in this region. Factors impeding females' access to cataract surgery should be taken into account when planning blindness prevention programs, and CSC targets should be relative to gender. More efforts are needed to increase the utilization of eye care services in South Asia, and especially the female population's access to cataract surgery.

## Figures and Tables

**Table 1:** Characteristics of studies included in the meta-analysis.

First author	Published year	Country	Design	Survey year	Location type	No. examined	Age group	Response rate (%)
Guruprasad et al. [19]	2013	India	PBCSS, RAAB	2011	Subnational, both	2907	≥50	95.30
Shaikh and Aziz [20]	2005	Pakistan	PBCSS, RAAB	2003	Subnational, rural	1076	≥50	93.60
Lepcha et al. [21]	2013	Bhutan	PBCSS, RAAB	2009	Subnational, both	4046	≥50	98.70
Thapa et al. [22]	2011	Nepal	PBCSS	After 2001	Subnational, urban	4003	≥40	83.39
Sherchan et al. [23]	2010	Nepal	PBCSS	2006	Subnational, rural	5138	≥50	86.80
Sapkota et al. [24]	2010	Nepal	PBCSS, RAAB	2006	Subnational, rural	4717	≥50	85.30
Murthy et al. [25]	2009	India	PBCSS	2007	Subnational, both	4738	≥50	91.90
Jadoon et al. [26]	2007	Pakistan	PBCSS	2002	Subnational, both	16570	≥30	95.50
Anjum et al. [27]	2006	Pakistan	PBCSS, RAAB	2004	Subnational, rural	1549	≥50	96.80
Sapkota et al. [28]	2006	Nepal	PBCSS, RAAB	2002	Subnational, rural	5002	≥45	85.30
Wadud et al. [29]	2006	Bangladesh	PBCSS, RAAB	2005	Subnational, rural	4868	≥50	91.90
Haider et al. [30]	2003	Pakistan	PBCSS, RAAB	2000	Subnational, both	1505	≥50	94.10
Nirmalan et al. [31]	2003	India	PBCSS	2000	Subnational, both	15265	≥50	92.30
Khan and Jadoon [32]	2015	Pakistan	PBCSS, RAAB	2015	Subnational, urban	3084	≥50	96.80
Sapkota and Limburg [33]	2012	Nepal	PBCSS, RAAB	2006–2010	National	39908	≥50	92.20
Muhit et al. [34]	2016	Bangladesh	PBCSS, RAAB	2010–2012	Subnational, both	21596	≥50	86.70

PBCSS: population-based cross-sectional study; RAAB: rapid assessment of avoidable blindness or rapid assessment of cataract surgical service.

**Table 2:** Risk of bias of individual studies.

First author	Published year	External validity				Internal validity						Summary item
		1	2	3	4	5	6	7	8	9	10	
Guruprasad et al. [19]	2013	Yes	Yes	Yes	Yes	Yes	Yes	Yes	Yes	Yes	Yes	Low risk of bias
Shaikh and Aziz [20]	2005	No	Yes	Yes	Yes	Yes	Yes	Yes	Yes	Yes	Yes	Low risk of bias
Lepcha et al. [21]	2013	Yes	Yes	Yes	Yes	Yes	Yes	Yes	Yes	Yes	Yes	Low risk of bias
Thapa et al. [22]	2011	No	Yes	Yes	Yes	Yes	Yes	Yes	Yes	Yes	Yes	Low risk of bias
Sherchan et al. [23]	2010	No	Yes	Yes	Yes	Yes	Yes	Yes	Yes	Yes	Yes	Low risk of bias
Sapkota et al. [24]	2010	No	Yes	Yes	Yes	Yes	Yes	Yes	Yes	Yes	Yes	Low risk of bias
Murthy et al. [25]	2009	Yes	Yes	Yes	Yes	Yes	Yes	Yes	Yes	Yes	Yes	Low risk of bias
Jadoon et al. [26]	2007	Yes	Yes	Yes	Yes	Yes	Yes	Yes	Yes	Yes	Yes	Low risk of bias
Anjum et al. [27]	2006	No	Yes	Yes	Yes	Yes	Yes	Yes	Yes	Yes	Yes	Low risk of bias
Sapkota et al. [28]	2006	No	Yes	Yes	Yes	Yes	Yes	Yes	Yes	Yes	Yes	Low risk of bias
Wadud et al. [29]	2006	No	Yes	Yes	Yes	Yes	Yes	Yes	Yes	Yes	Yes	Low risk of bias
Haider et al. [30]	2003	Yes	Yes	Yes	Yes	Yes	Yes	Yes	Yes	Yes	Yes	Low risk of bias
Nirmalan et al. [31]	2003	Yes	Yes	Yes	Yes	Yes	Yes	Yes	Yes	Yes	Yes	Low risk of bias
Khan and Jadoon [32]	2015	No	Yes	Yes	Yes	Yes	Yes	Yes	Yes	Yes	Yes	Low risk of bias
Sapkota and Limburg [33]	2012	Yes	Yes	Yes	Yes	Yes	Yes	Yes	Yes	Yes	Yes	Low risk of bias
Muhit et al. [34]	2016	Yes	Yes	Yes	Yes	Yes	Yes	Yes	Yes	No	Yes	Low risk of bias

1 = was the study's target population a close representation of the national population in relation to relevant variables? 2 = was the sampling frame a true or close representation of the target population? 3 = was some form of random selection used to select the sample, OR; was a census undertaken? 4 = was the likelihood of nonresponse bias minimal? 5 = were data collected directly from the subjects? 6 = was an acceptable case definition used in the study? 7 = was the study instrument that measured the parameter of interest shown to have reliability and validity? 8 = was the same mode of data collection used for all subjects? 9 = was the length of the shortest prevalence period for the parameter of interest appropriate? 10 = were the numerator and denominator for the parameter of interest appropriate?

**Table 3:** Cataract surgical coverage (CSC) in male and female at different visual acuity level.

First author	Published year	CSC (person, VA <3/60)			CSC (person, VA <6/18)			CSC (eyes, VA <3/60)			CSC (eyes, VA <6/18)		
		Male	Female	OR	Male	Female	OR	Male	Female	OR	Male	Female	OR
Guruprasad et al. [19]	2013	84.6	79.7	1.4	NA	NA	—	72.1	67.8	1.2	NA	NA	—
Shaikh and Aziz [20]	2005	NA	NA	—	NA	NA	—	51.9	47.8	1.2	NA	NA	—
Lepcha et al. [21]	2013	77.8	67.7	1.7	NA	NA	—	61.5	51.3	1.5	NA	NA	—
Thapa et al. [22]	2011	85.3	93.9	0.4	NA	NA	—	NA	NA	—	NA	NA	—
Sherchan et al. [23]	2010	61.7	70.8	0.7	NA	NA	—	NA	NA	—	NA	NA	—
Sapkota et al. [24]	2010	39.3	35.8	1.2	NA	NA	—	NA	NA	—	NA	NA	—
Murthy et al. [25]	2009	75.5	69.7	1.3	NA	NA	—	NA	NA	—	NA	NA	—
Jadoon et al. [26]	2007	79.6	74.9	1.3	44.6	42.8	1.1	64.5	58.4	1.3	42.8	38.6	1.2
Anjum et al. [27]	2006	68.9	51.6	2.1	NA	NA	—	49.5	41.3	1.4	NA	NA	—
Sapkota et al. [28]	2006	68.1	52.6	1.9	NA	NA	—	NA	NA	—	NA	NA	—
Wadud et al. [29]	2006	63.5	59.0	1.2	34.5	36.4	0.9	34.6	34.9	1.0	17.4	18.7	0.9
Haider et al. [30]	2003	92.8	73.9	4.6	53.3	45.5	1.4	75.7	63.7	1.8	39.1	33.1	1.3
Nirmalan et al. [31]	2003	74.4	60.5	1.9	NA	NA	—	67.7	46.8	2.4	NA	NA	—
Khan and Jadoon [32]	2015	92.5	92.6	1.0	75.5	71.5	1.2	77.0	75.2	1.1	62.0	54.0	1.4
Sapkota and Limburg [33]	2012	87.6	82.9	1.5	55.7	53.5	1.1	68.9	65.7	1.2	40.0	38.8	1.1
Muhit et al. [34]	2016	76.6	64.3	1.8	35.1	30.5	1.2	61.5	49.7	1.6	20.1	21.3	0.9
Overall weighted mean			**1.5**			**1.1**			**1.4**			**1.1**	

OR, odds ratio; NA, not available; VA, visual acuity.

## Appendix

### 1. Search Strategy

1. “Cataract Extraction” [Mesh]
2. “Phacoemulsification” [Mesh]
3. “Lens Implantation, Intraocular” [Mesh]
4. “Phakic Intraocular Lenses” [Mesh]
5. Phacoemulsification [Title/Abstract]
6. “Extracapsular cataract extraction” [Title/Abstract]
7. “Manual small incision cataract surgery” [Title/Abstract]
8. Phaco [Title/Abstract] OR Phako [Title/Abstract]
9. ECCE [All Fields] OR MSICS [All Fields] OR SICS [All Fields]
10. Intraocular lens*∗*[All Fields] OR intraocular lens*∗*[All Fields] OR IOL*∗*[All Fields]
11. “cataract surgical coverage” [All Fields]
12. “cataract surgery coverage” [All Fields]
13. CSC [All Fields]
14. #1OR #2 OR #3 OR #4 OR #5 OR #6 OR #7OR #8 OR #9 OR #10 OR #11 OR #12 OR #13
15. “Sex Factors” [Mesh]
16. Gender [All Fields]
17. Female [All Fields]
18. Sex [All Fields]
19. #15 OR #16 OR #17 OR #18
20. “Epidemiology” [Mesh]
21. “Cross-Sectional Studies” [Mesh]
22. Epidemiology [All Fields]
23. “population-based study” [All Fields]
24. #20 OR #21 OR #22 OR #23
25. South Asia [All Fields]
26. India [All Fields] OR Pakistan [All Fields] OR Bangladesh [All Fields] OR Maldives [All Fields] OR Sri Lanka [All Fields] OR Bhutan [All Fields] OR Nepal [All Fields]
27. #25 OR #26
28. #14 AND #19 AND #24 AND #27
